# A good response to anti-PD-1 monoclonal antibody plus SBRT in a patient with PD-L1-negative recurrent advanced esophageal cancer: a long-term follow-up case report of a possible abscopal effect

**DOI:** 10.3389/fonc.2024.1369035

**Published:** 2024-06-27

**Authors:** Tao Hai, Jia Liu, Jialu Lai, Lin Zhou

**Affiliations:** ^1^ Division of Thoracic Tumor Multimodality Treatment, Cancer Center and State Key Laboratory of Biotherapy, West China Hospital, Sichuan University, Chengdu, Sichuan, China; ^2^ Department of Oncology, Chengdu First People’s Hospital, Chengdu, Sichuan, China; ^3^ Department of Radiation Oncology, Cancer Center and State Key Laboratory of Biotherapy, West China Hospital, Sichuan University, Chengdu, Sichuan, China

**Keywords:** abscopal effect, anti-PD-1 monoclonal antibody, stereotactic body radiotherapy, esophageal cancer, case report

## Abstract

There are limited treatment options for recurrent advanced esophageal squamous cell carcinoma. A good response with a possible abscopal effect was observed in a patient with programmed death-ligand 1 (PD-L1)-negative recurrent advanced esophageal squamous cell carcinoma treated with an anti-PD-1 monoclonal antibody plus stereotactic body radiotherapy (SBRT). A 66-year-old male patient was diagnosed with recurrent advanced esophageal squamous cell carcinoma with multiple lung metastases (13 metastatic nodules in total) four months after completing radical radiotherapy plus concurrent and consolidated chemotherapy, and PD-L1 expression in the primary esophageal tumor was negative. This patient received 25 cycles of camrelizumab (an anti-PD-1 monoclonal antibody) in total plus upfront SBRT for two metastatic nodules, which was administered after the first cycle of camrelizumab. After this combined treatment, for most nontarget nodules, an obvious volume decrease and fuzzy change were observed, including two nodules that completely vanished. At the end of follow-up, the progression-free survival and duration of response of this patient were 34 months and 32 months, respectively. This case report indicated that an anti-PD-1 monoclonal antibody combined with SBRT was a promising therapeutic strategy for recurrent esophageal squamous cell carcinoma even in patients with negative PD-L1 expression.

## Introduction

The abscopal effect is a rare and intriguing phenomenon that has been reported in many cancers treated with radiotherapy, especially stereotactic body radiotherapy (SBRT) and hypofractionated radiotherapy (HFRT). This phenomenon is defined as regression in distant nonirradiated metastatic tumor sites after local irradiation for targeted lesion, and may be caused by the tumor-specific immune response of T cells to tumor-associated antigens triggered by the radiation of target lesions (1). There are only a few case reports regarding the abscopal effect in advanced esophageal cancer patients, which were triggered by palliative radiotherapy at a dose of 30 Gy in 10 fractions to the primary tumor and/or mediastinal lymph nodes after the failure of chemotherapy (2, 3), at a dose of 40 Gy in 10 fractions to the metastatic cervical lymph node after the failure of immunotherapy (4), and by SBRT at 42 Gy in 6 fractions to the metastatic retroperitoneal lymph node after the failure of chemoimmunotherapy (5). Generally, the abscopal effect is quite rare in patients who receive radiotherapy alone; however, several reports have indicated that radiotherapy combined with immune checkpoint inhibitors (ICIs) might increase the incidence of an abscopal effect (1, 6–9).

In this case report, the possible abscopal effect was described in a programmed death-ligand 1 (PD-L1)-negative recurrent esophageal squamous cell carcinoma patient with multiple lung metastases who was treated with an anti-PD-1 monoclonal antibody plus stereotactic body radiotherapy (SBRT). Informed consent was signed, and ethical approval was obtained from our institutional review board.

## Case report

**Figure 1:**
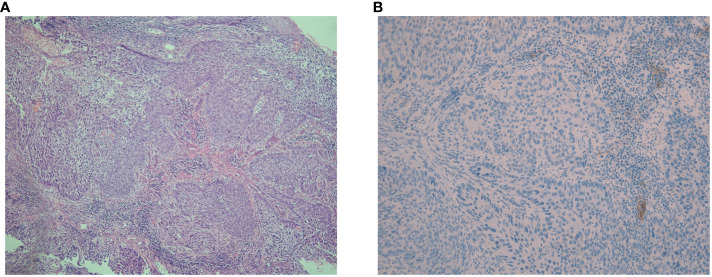
Hematoxylin-eosin and immunohistochemical staining for PD-L1 expression in pathological sections. **(A)** The hematoxylin-eosin staining of pathological sections revealed poorly differentiated squamous cell carcinoma with heterotypic cells arranged in nests, interstitial infiltration, and no obvious keratosis (magnification, ×100). **(B)** The immunohistochemical PD-L1 expression test using Dako’s 22C3 assay for the primary esophageal tumor showed a tumor proportion score of 0 with a combined positive score of <1 (magnification, ×200).

A 66-year-old male patient was diagnosed with upper thoracic esophageal poorly differentiated squamous cell carcinoma (cT3N2M0, stage III) (**Figure 1A**) in a local hospital in June 2020 by esophagoscopy biopsy, chest and upper abdominal computed tomography (CT), and an esophageal barium meal. His past medical history was unremarkable except for more than 10 years of hypertension and more than 40 years of smoking and alcohol consumption. From July 2020 to December 2020, intensity-modulated radiotherapy, with a dose of 66 Gy/33 fractions for the primary tumor and involved regional lymph nodes, plus concurrent and consolidated chemotherapy with six cycles of docetaxel combined with cisplatin were delivered to the patient, and a partial response was achieved. However, four months later (April 2021), routine thoracic CT revealed multiple metastatic nodules in the lungs.

**Figure 2:**
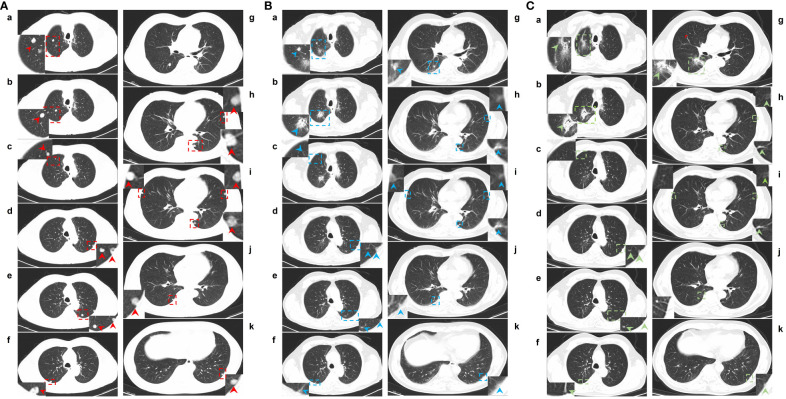
CT images of lung metastases at baseline and after two cycles and 18 cycles of camrelizumab plus SBRT. **(A)**, a-k: Lung window of CT images for lung metastases at baseline. **(B)**, a-k: Lung windows of CT images of lung metastases after 2 cycles of camrelizumab plus SBRT. **(C)**, a-k: Lung windows of CT images for lung metastases after 18 cycles of camrelizumab plus SBRT. In total, 13 metastatic nodules were located in the lungs. One nodule located in the right upper lung (Ab) and one in the right lower lung (Ag) received SBRT at 45 Gy/5 f, one day for each alternate, twelve days after the first cycle of camrelizumab. The second cycle of camrelizumab was administered 2 weeks after SBRT, and repeat thoracic CT was delivered 4 weeks later. On the repeat CT scan, the metastases that received SBRT showed fibrosis, fuzziness, and consolidation changes (Bb and Bg). For most metastases that did not receive SBRT, an obvious volume decrease and fuzzy change were observed, including one nodule that completely vanished (Bc). Other 16 cycles of camrelizumab were administered; the repeat CT showed more obvious fibrosis, fuzziness, and consolidation changes in the target nodules, and the nontarget nodule received radiation at a relatively high dose (Ca and Cf). The volume of other nontarget nodules continued to decrease, and the fuzzy change was more obvious, including another nodule that completely vanished (Cj). Red, blue, and green arrows: metastases located in the lungs at baseline and after treatment. Red and blue dotted boxes: particular areas surrounding metastases, which are magnified and displayed on the side.

**Figure 3:**
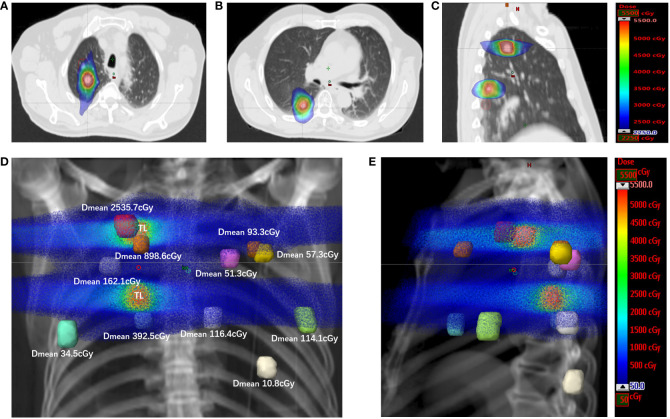
The spatial dose distribution for target and nontarget metastases of the SBRT plan. **(A)** The dose distribution for the target metastasis located in the right upper lung, with displayed isodoses ranging from 2250 cGy to 5500 cGy. **(B)** The dose distribution for the target metastasis located in the right lower lung, with the displayed isodoses ranging from 2250 cGy to 5500 cGy. **(C)** The dose distributions for target metastases in sagittal sections, with the displayed isodoses ranging from 2250 cGy to 5500 cGy. **(D)** The spatial dose distribution for target and nontarget metastases in the DDR coronal plane. Each colored nodule represents the planning volume of different target or nontarget metastases (uniformly expanded 5 mm for metastases), and the mean dose for the planning volume of each nontarget metastasis is displayed on the side (D_mean_). The displayed isodoses ranged from 50 cGy to 5500 cGy. TL, target lesion; DDR, direct digital radiography. **(E)** The spatial dose distribution for target and nontarget metastases in the DDR sagittal plane. Each colored nodule represents the planning volume of different target or nontarget metastases. The displayed isodoses ranged from 50 cGy to 5500 cGy. TL, target lesion; DDR, direct digital radiography.

**Figure 4:**
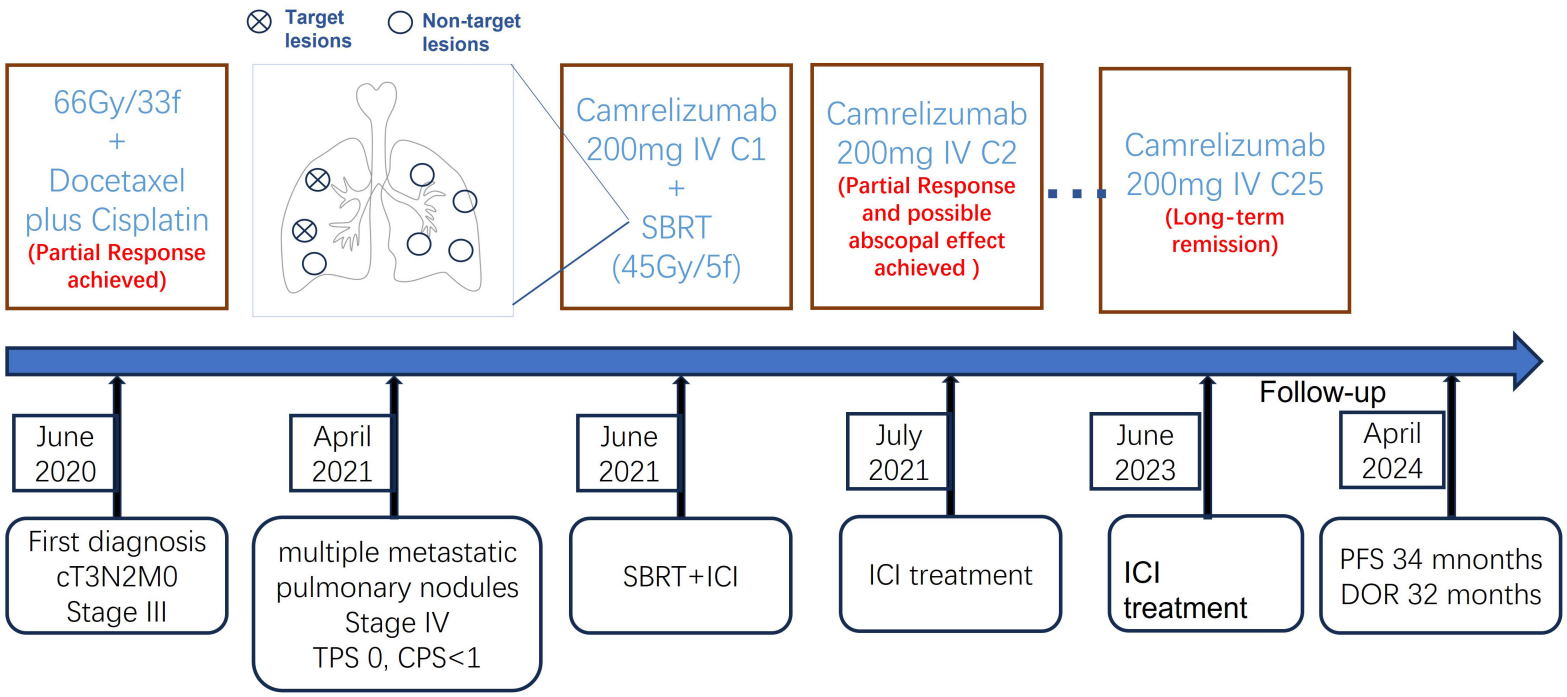
The clinical course and timeline of the present case. TPS, tumor proportion score; CPS, combined positive score; IV, intravenous; SBRT, stereotactic body radiotherapy; ICI, immune checkpoint inhibitor; PFS, progression-free survival; DOR, duration of response.

In May 2021, this patient was transferred to our hospital. The immunohistochemical PD-L1 expression test using Dako’s 22C3 assay for the primary esophageal tumor showed a tumor proportion score (TPS, which is calculated as the number of PD-L1 positive tumor cells divided by the total number of all tumor cells multiplied by 100) of 0 with a combined positive score (CPS, which is calculated as the number of PD-L1 positive cells, including tumor cells, lymphocytes, and macrophages divided by the total number of viable tumor cells multiplied by 100) of <1 (**Figure 1B**). The baseline thoracic CT revealed a slightly thickened esophageal wall and 13 metastatic nodules with diameters ranging from 7 mm to 11 mm (**Figures 2A**a–k) in the bilateral lungs. Other imaging examinations, including abdominal CT, brain magnetic resonance imaging, and bone single-photon emission computed tomography, revealed no other metastases. On June 16^th^, 2021, the first cycle of camrelizumab (200 mg IV) was administered on day 1. Twelve days later, SBRT (**Figures 3A–C**) was delivered to two nodules located in the right upper and lower lung (**Figures 2A**b, g), and the prescribed dose was 45 Gy in 5 fractions, which was given every other day for each nodule alternately. The second cycle of camrelizumab (200 mg IV) on day 1 was administered 2 weeks after SBRT. Four weeks later, the repeat thoracic CT showed fibrosis, fuzziness, and consolidation changes in the target nodules (**Figures 2B**b, g). For most of the nontarget nodules, obvious volume decreases and fuzzy changes were observed (**Figures 2B**a–k), including one nodule that completely vanished (**Figure 2B**c). From August 2021 to June 2023, this patient received another 23 cycles of camrelizumab, 200 mg IV on day 1, every 3-4 weeks (25 cycles in total), and thoracic and upper abdominal CT scans were taken every 3 to 4 months regularly with no oncotherapy thereafter. On December 2022, after 18 cycles of camrelizumab, repeated thoracic CT showed more obvious fibrosis, fuzziness, and consolidation changes in the target nodules, and the nontarget nodules received radiation at a quite high dose (**Figures 2C**a, b, g). The volume of other nontarget nodules continued to decrease, and the fuzzy change was more obvious (**Figures 2C**b–k), including another nodule that completely vanished (**Figure 2C**j). Regular CT scans after December 2022 showed no disease progression. At the end of follow-up on April 2024, the progression-free survival and duration of response of this patient were 34 months and 32 months, respectively. The baseline peripheral blood CD8+ and CD4+ T lymphocyte counts were 124 cells/μL and 263 cells/μL, respectively, compared to 122 cells/μL and 156 cells/μL after the second cycle of camrelizumab and 148 cells/μL and 271 cells/μL after the 6^th^ cycles of camrelizumab, respectively, and there was no subsequent lymphocyte test thereafter. The clinical course and timeline of the present case are shown in **Figure 4**.

## Discussion

There are limited treatment options for patients with recurrent advanced esophageal squamous cell carcinoma. In addition, all of these treatments showed unsatisfactory efficacy. Pembrolizumab has been proven to be superior to chemotherapy as a second-line therapy for advanced esophageal cancer patients with a PD-L1 CPS≥10 (10). However, the objective response rate of pembrolizumab for esophageal squamous cell carcinoma patients with a PD-L1 CPS<10 was only 11.9% (10). Camrelizumab, manufactured by Jiangsu Hengrui Pharmaceuticals Co., Ltd., China, is an anti-PD-1 monoclonal antibody that has been demonstrated to be superior to chemotherapy for recurrent advanced esophageal squamous cell carcinoma patients regardless of PD-L1 expression. However, in patients with PD-L1 < 1%, camrelizumab seemed to be less effective and not superior to chemotherapy (11). PD-L1 expression has also been demonstrated as an available predictor for the efficacy of anti-PD-1 monoclonal antibodies for other tumor types, and patients with low PD-L1 expression have limited efficacy from anti-PD-1 monoclonal antibodies (12). Furthermore, it has been reported that PD-L1 expression and tumor-associated inflammatory infiltrating cells in metastases are even lower than those in primary tumors in patients with esophageal cancer (13). The PD-L1 expression in the primary tumor in the present patient was negative; therefore, the good response of the lung metastases was more likely due to the abscopal effect induced by SBRT in the setting of camrelizumab treatment.

The abscopal effect is a fascinating phenomenon that is occasionally induced by SBRT alone or combined with ICIs. SBRT seems to be one of the optimal radiation treatment modalities for inducing antitumor immunity, and the synergistic effect of ICIs combined with SBRT has been demonstrated by several studies (1, 6–8). However, there are very limited reports on the therapeutic effect of this combined treatment for esophageal cancer (2–5). To the best of our knowledge, the present study is the first case report of a possible abscopal effect observed in a PD-L1-negative advanced esophageal cancer patient treated with an anti-PD-1 monoclonal antibody plus SBRT.

In addition to direct tumor cell killing by deoxyribonucleic acid damage, radiotherapy can also elicit immune-mediated antitumor responses by releasing tumor neoantigens reprogramming the tumor microenvironment to increase the recruitment and function of antigen-presenting cells and T cells, and releasing cytokines and chemokines, such as interferons, interleukins, and transforming growth factor beta (1). The tumor neoantigens released from irradiated tumors are taken up by antigen-presenting cells and presented to T cells in the lymph nodes via the MHC pathway. Then, activated CD8+ T cells exit the lymph nodes and home to primary tumors and nonirradiated metastases, which may result in an “abscopal effect” (1). However, because of the insufficient ability of radiotherapy alone to overcome the immune tolerance mechanisms of tumor cells, reports of abscopal effects due to radiotherapy alone are rare. Several studies have indicated that radiotherapy combined with ICIs might overcome tumor immunosuppression and increase the incidence of an abscopal effect (6–9). Recently, many reports have shown the encouraging synergistic effect of conventional chemoradiotherapy combined with anti-PD1 monoclonal antibodies in patients with resectable or locally advanced esophageal cancer (14, 15), yet there are no published prospective data about the abscopal effect of radiotherapy combined with ICI treatment in patients with metastatic esophageal cancer. However, in advanced NSCLC, studies have shown that the combination of ICIs and stereotactic ablative radiotherapy (SABR) for a single lesion located in the lung could improve local control and overall survival compared to ICIs alone (16, 17). Furthermore, due to the heterogeneity of tumor lesions, SBRT targeting multiple lesions can generate a wider spectrum of tumor antigens, thereby improving the synergistic effect with immunotherapy. Therefore, multitarget SBRT may be a more appropriate treatment strategy when combined with ICIs (18). Furthermore, considering that a radiation dose of 8-12 Gy per fraction seems to be more effective in inducing abscopal effects than lower/higher doses per fraction (19, 20), a dose of 9 Gy per fraction was prescribed for two separate lesions in the present case.

Low-dose radiotherapy (LDRT) can reverse tumor immune desertification and resistance to immunotherapy, and scattered low-dose irradiation might improve the response of metastases to immunotherapy when combined with high-dose radiation to another lesion (21–23). In a preclinical study reported by Yin et al., LDRT could amplify the systemic effect of HFRT by increasing secretion of chemokines involved in the attraction of T cells, and inducing recruitment of CD4+ T cells, CD8+ T cells, and dendritic cells (23). Compared to single and double combined treatment, triple combined treatment with HFRT at a dose of 24 Gy in 3 fractions delivered to the primary tumor, LDRT at a dose of 2 Gy in 1 fraction delivered to the abscopal tumor, and systematic treatment with anti-mouse PD1 resulted in the greatest tumor response and longest survival, with most robust infiltration of CD8+ effector T cells and the lowest infiltration of myeloid-derived suppressor cells (MDSCs) in the abscopal tumor (23). In the present case, two nontarget metastases near the target nodule received a quite high dose of radiation (red one in the upper right lung, mean dose of 2535.7 cGy; **Figures 3D**, **2A**a, **B**a, **C**a; aurantium one in the upper right lung, mean dose of 898.6 cGy; **Figures 3D**, **2A**f, **B**f, **C**f), and most of the other nontarget metastases received a quite low dose of radiation (mean dose of 34.5-392.5 cGy; **Figures 3D, E**) via exposure to the entrance and/or exit rays of SBRT, except for one patient who received a scattered dose of 10.8 cGy (**Figures 2A**k, **B**k, **C**k, **3D, E**). The low dose of radiation to nontarget metastases might be one reason for the good response to the combined treatment.

ICIs is one of the treatment options for recurrent advanced esophageal squamous cell carcinoma. However, mono-ICI treatment can only have limited efficacy for patients with negative or low PD-L1 expression (10, 11). According to previous reports and the present case, additional upfront SBRT may improve the efficacy of ICI treatment. Considering that LDRT may enhance the synergetic effect of ICI treatment combined with SBRT, the target lesion of SBRT should be chosen as the scattered radiation dose can cover most of the remaining lesions. Furthermore, if possible, it is advisable to select multiple lesions as targets for SBRT, given the heterogeneous nature of the tumor.

There were many limitations of the present case. The major limitation was that there was no evaluation after the first cycle of camrelizumab treatment and before SBRT for this patient and that no PD-L1 expression was detected in the metastases; therefore, it was unclear whether the good response of the metastatic tumors was due to ICI treatment, SBRT, or their combination. The other limitations included a lack of genomic and transcriptomic profiling data, a lack of sequential CD8+ and CD4+ T lymphocyte detection in peripheral blood, and a lack of biopsy specimens from metastatic lesions to detect the tumor microenvironment and lymphocyte infiltration. Preclinical and clinical studies are warranted to further reveal the underlying mechanisms of the synergetic effect of ICI treatment and radiotherapy and the optimal strategy for this combination.

## Conclusion

The present case indicated that the use of an anti-PD-1 monoclonal antibody combined with SBRT might be a promising therapeutic strategy for recurrent esophageal squamous cell carcinoma even in patients with negative PD-L1 expression. The optimal schedule of combined treatment and underlying mechanisms are worthy of further exploration.

## Data availability statement

The original contributions presented in the study are included in the article/supplementary material. Further inquiries can be directed to the corresponding author/s.

## Ethics statement

The studies involving humans were approved by Institutional Review Board of the West China Hospital, Sichuan University. The studies were conducted in accordance with the local legislation and institutional requirements. The participants provided their written informed consent to participate in this study. Written informed consent was obtained from the individual(s) for the publication of any potentially identifiable images or data included in this article.

## Author contributions

TH: Writing – original draft, Writing – review & editing. JL: Writing – review & editing. JLL: Writing – review & editing. LZ: Writing – review & editing.
